# Supplementary material for: Innovative infrastructure to access Brazilian fungal diversity using deep learning
Source: PeerJ. 2024 Jul 9;12:e17686. doi: 10.7717/peerj.17686 (PMC11243970; doi:10.7717/peerj.17686)
Supplement: Supplemental Information 3 [file peerj-12-17686-s003.pdf]

INCLUSION of Dr. Emerson L. Gumboski as one of the authors

Belo Horizonte-MG-Brazil
May 14th, 2024

Dear Academic Editor (***PeerJ)*** and Reviewers:

We understand that the addition of a new author to a submission which has already had a first decision is a rare event. Nonetheless, when we submitted the first version, Dr. E. Ricardo Drechsler-Santos, the last author of our paper and leader of the group of Macrofungal Conservation and Taxonomy, was moving from Brasil to UF (University of Florida, U.S.A.) with all his familly (including his two small children) to U.S.A. for a sabbatical year and, due to all of this big change in his life, he completely forgot and inadvertently did not include our colleague Dr. Emerson L. Gumboski (UNIVILLE, Brazil) as one of the co-authors.

Dr. Emerson L. Gumboski (UNIVILLE, Brazil) is the only specialist in lichenized fungi in our group. A total of 48 lichen species in our databese (almost comprising 10% of the total of 505 macrofungal species) was personally collected, in different biomes, by him, who processed the material, generate the field photographs, and identified all the collected specimens at species level. Furthermore, for this new version of our manuscript, I asked for him to perform the analyses that resulted in the (new) Figure 3, as well as to construct the (new) Figures 1 and 2. Furthermore, he reviewed all this new version and ALL the authors fully understood and formally accepted his inclusion, as you can check in PeerJ system submission.

Sincerely (on behalf of all the authors),

Prof. Dr. Aristóteles Góes-Neto
Universidade Federal de Minas Gerais (UFMG)
Av. Antônio Carlos, 6627, Pampulha, Belo Horizonte, MG, Brazil, CEP 31270-901
E-mails: arigoesneto@icb.ufmg.br / arigoesneto@gmail.com
CV: http://lattes.cnpq.br/6134133834289438
ORCID: 0000-0002-7692-6243
